# 1350. Solid Organ Transplantation From COVID Positive Donors: Trends in Utilization, Discard & Long-term Outcomes in the United States

**DOI:** 10.1093/ofid/ofad500.1187

**Published:** 2023-11-27

**Authors:** Abhay Dhand, Kenji Okumura, Suguru Ohira, Steven Lansman, Seigo Nishida

**Affiliations:** Westchester Medical Center, Valhalla, NY, Valhalla, NY; Westchester Medical Center, Valhalla, NY, Valhalla, NY; Westchester Medical Center, Valhalla, NY, Valhalla, NY; Westchester Medical Center, Valhalla, NY, Valhalla, NY; Westchester Medical Center, Valhalla, NY, Valhalla, NY

## Abstract

**Background:**

Transplantation of organs from COVID positive (COVID+) donors is increasing. The aim of this study was to assess the trends in utilization, discard, and longer-term outcomes in solid organ transplant (SOT) recipients who received organs from COVID+ donors in the United States (US).

**Methods:**

Rates of utilization, discard, and outcomes of SOT from deceased donors with a positive COVID PCR test from respiratory tract between March 2020 and December 2022 were analyzed using the de-identified United Network for Organ Sharing (UNOS) database.

**Results:**

During the study period, 1185 COVID+ donors led to the transplantation of 1249 kidneys, 592 livers, and 168 hearts.

The center-wise acceptance rate for organs from COVID+ donors increased from 2021 to 2022: heart from 31% to 73%, kidney from 53% to 88%, and liver from 53% to 89%.

Discard rates of kidneys from COVID+ donors remained high: left kidney- 29%, right kidney- 32% and were significantly higher than kidney discard rates for COVID negative (COVID-) donors (p < 0.001)

When compared to COVID- donors, COVID+ donors were younger and had a lower median Kidney Donor Profile Index (0.51 vs. 0.54, *p* = .004), lower median serum creatinine (0.9 vs. 1.05 mg/dl, *p<* 0.001), similar median serum total bilirubin (0.6 mg/dl, *p* = .15), and similar left ventricular ejection fraction (60%, *p* = .84). Six months, one-year and 18-month overall and graft survival were comparable between recipients of COVID+ and COVID- donors (table 1) (figures 1).

**Table 1:**
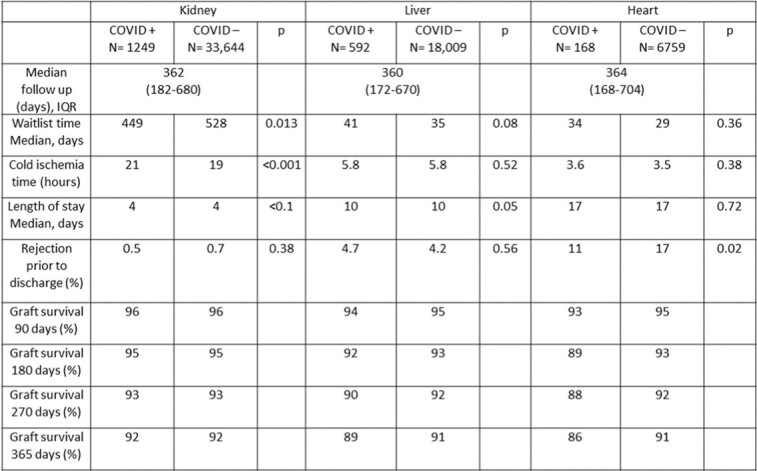
Characteristics and outcomes of solid organ transplantation from COVID+ and COVID- donors

Graft Survival: COVID+ vs. COVID- donors

**Figure:**
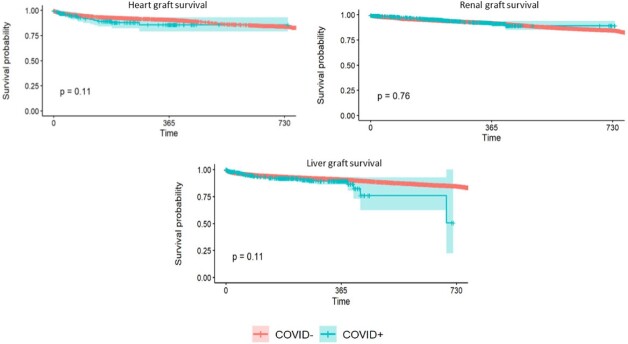

**Conclusion:**

Utilization of organs from COVID+ donors has improved across various transplant centers in the US. Kidney discard rate from COVID+ donors remain high. Longer-term outcomes of SOT from COVID+ donors are encouraging and are helping to successfully expand the donor pool.

**Disclosures:**

**All Authors**: No reported disclosures

